# Target-agnostic drug prediction integrated with medical record analysis uncovers differential associations of statins with increased survival in COVID-19 patients

**DOI:** 10.1371/journal.pcbi.1011050

**Published:** 2023-05-05

**Authors:** Megan M. Sperry, Tomiko T. Oskotsky, Ivana Marić, Shruti Kaushal, Takako Takeda, Viktor Horvath, Rani K. Powers, Melissa Rodas, Brooke Furlong, Mercy Soong, Pranav Prabhala, Girija Goyal, Kenneth E. Carlson, Ronald J. Wong, Idit Kosti, Brian L. Le, James Logue, Holly Hammond, Matthew Frieman, David K. Stevenson, Donald E. Ingber, Marina Sirota, Richard Novak

**Affiliations:** 1 Wyss Institute for Biologically Inspired Engineering, Harvard University, Boston, Massachusetts, United States of America; 2 Department of Biology, Tufts University, Medford, Massachusetts, United States of America; 3 Bakar Computational Health Sciences Institute, University of California San Francisco, San Francisco, California, United States of America; 4 Department of Pediatrics, University of California San Francisco, San Francisco, California, United States of America; 5 Department of Pediatrics, School of Medicine, Stanford University, Stanford, California, United States of America; 6 Center for Academic Medicine, Stanford University School of Medicine, Stanford, California, United States of America; 7 Department of Microbiology and Immunology, University of Maryland School of Medicine, Baltimore, Maryland, United States of America; 8 Vascular Biology Program and Department of Surgery, Boston Children’s Hospital and Harvard Medical School, Boston, Massachusetts, United States of America; 9 Harvard John A. Paulson School of Engineering and Applied Sciences, Cambridge, Massachusetts, United States of America; National Center for Biotechnology Information (NCBI), UNITED STATES

## Abstract

Drug repurposing requires distinguishing established drug class targets from novel molecule-specific mechanisms and rapidly derisking their therapeutic potential in a time-critical manner, particularly in a pandemic scenario. In response to the challenge to rapidly identify treatment options for COVID-19, several studies reported that statins, as a drug class, reduce mortality in these patients. However, it is unknown if different statins exhibit consistent function or may have varying therapeutic benefit. A Bayesian network tool was used to predict drugs that shift the host transcriptomic response to SARS-CoV-2 infection towards a healthy state. Drugs were predicted using 14 RNA-sequencing datasets from 72 autopsy tissues and 465 COVID-19 patient samples or from cultured human cells and organoids infected with SARS-CoV-2. Top drug predictions included statins, which were then assessed using electronic medical records containing over 4,000 COVID-19 patients on statins to determine mortality risk in patients prescribed specific statins versus untreated matched controls. The same drugs were tested in Vero E6 cells infected with SARS-CoV-2 and human endothelial cells infected with a related OC43 coronavirus. Simvastatin was among the most highly predicted compounds (14/14 datasets) and five other statins, including atorvastatin, were predicted to be active in > 50% of analyses. Analysis of the clinical database revealed that reduced mortality risk was only observed in COVID-19 patients prescribed a subset of statins, including simvastatin and atorvastatin. *In vitro* testing of SARS-CoV-2 infected cells revealed simvastatin to be a potent direct inhibitor whereas most other statins were less effective. Simvastatin also inhibited OC43 infection and reduced cytokine production in endothelial cells. Statins may differ in their ability to sustain the lives of COVID-19 patients despite having a shared drug target and lipid-modifying mechanism of action. These findings highlight the value of target-agnostic drug prediction coupled with patient databases to identify and clinically evaluate non-obvious mechanisms and derisk and accelerate drug repurposing opportunities.

## Author summary

Retrospective studies have shown that COVID-19 patients taking HMGCR-inhibiting statins exhibit a reduced risk of mortality. However, potential variability in statin effects has been detected and studies from diverse sub-fields also suggest that this seemingly homogeneous class of compounds may function differently despite close chemical similarity. Based on predictions of a computational drug repurposing algorithm in combination with clinical evidence gathered during the COVID-19 pandemic, we identified that different statin types appear to have different biological properties despite similar chemical structure and they vary in their association with increased survival in COVID-19 patients. Together, *in silico* predictions from patient transcriptomics data, *in vitro* assays of infection, cytotoxicity, and host response, and retrospective clinical data provide a more comprehensive assessment of individual statin activity. Our findings, along with other emerging data, suggest that some statins (i.e., simvastatin, atorvastatin, and rosuvastatin), but not others, have a mitigating effect on the severity of COVID-19 disease reflected in a reduction in mortality. Importantly, different statins seem to exert different biological activities despite similar chemical structure and shared known mechanism of action related to lipid metabolism. These findings suggest that drug repurposing efforts may require consideration of drug-specific effects rather than accepted mechanisms.

## Introduction

The emergence of the COVID-19 pandemic presented an urgent need for new and effective therapeutics, and repurposing of approved drugs with known safety profiles offered a path to identify viable treatment options. Research programs that focused on drug repurposing for COVID-19 presented opportunities to broadly uncover and understand new features of existing drugs [1–3]. Recent retrospective studies by members of our group and others have shown that COVID-19 patients taking drugs from one of the most prescribed drug classes in the world–statins–exhibit a reduced mortality rate, but these studies pooled all statin compounds (e.g., lovastatin, simvastatin, atorvastatin, etc.) together in their analyses [4–6]. All statins are prescribed to lower lipid and cholesterol levels, and share a common mechanism involving inhibition of HMG-CoA reductase (HMGCR); however, statins are also known to have anti-inflammatory and immunomodulatory properties, through mechanisms that involve several pathways [5–11], potentially by upregulating heme oxygenase-1 (HO-1) [7]. In addition, while three retrospective studies that pooled all statins demonstrated a significant reduction in mortality risk, no improvement in mortality outcome could be detected in another study [10]. This raises the possibility that different statins might differ in their ability to reduce morbidity and mortality in COVID-19 patients, which could influence the results of studies based on which drugs were included. Moreover, if true, it would be important to distribute this information widely because it could influence clinical decision-making with regards to statin selection during the current COVID-19 crisis [12,13].

**Fig 1:**
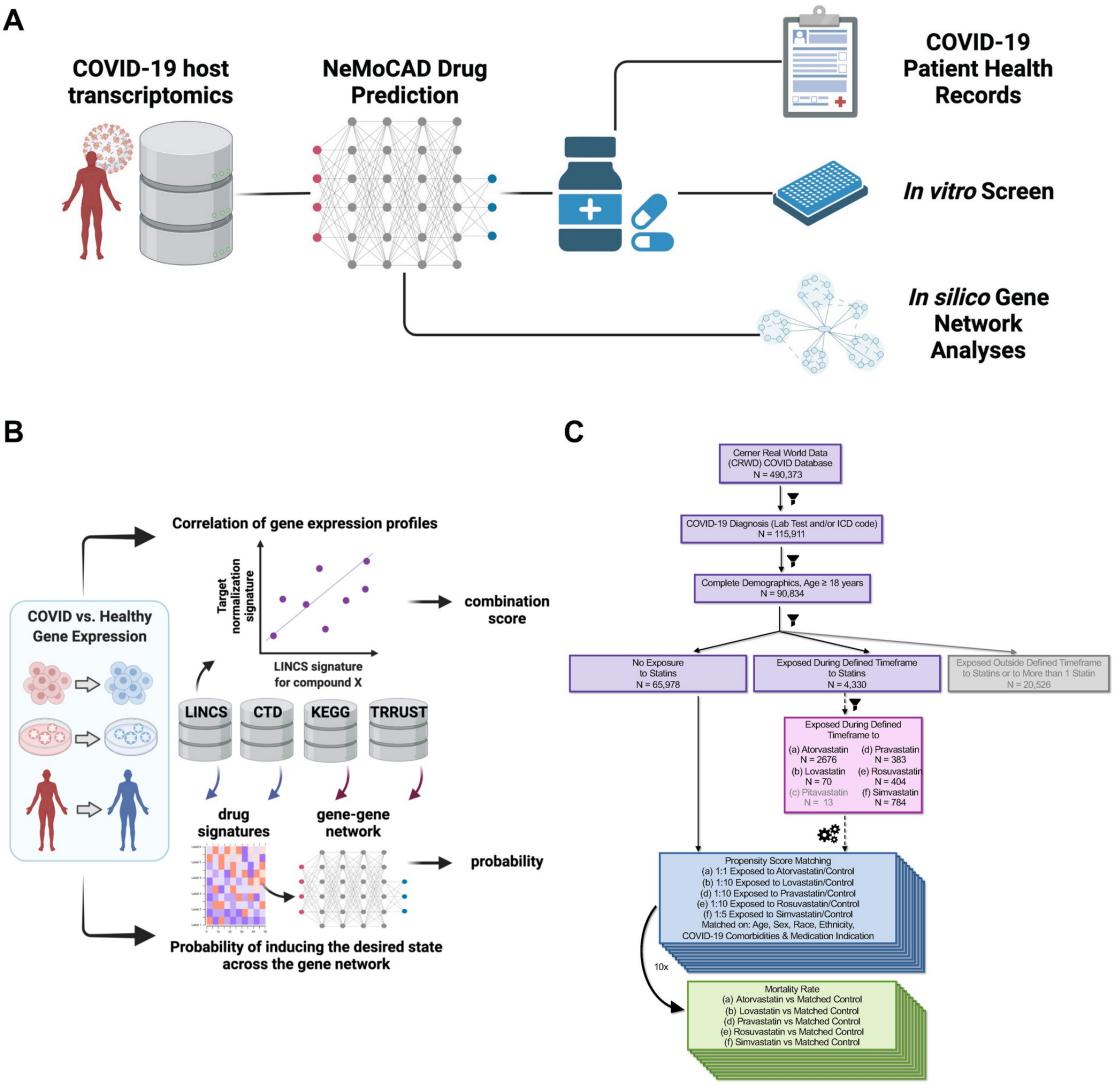
Combination approach for pathway-agnostic identification of compounds for drug repositioning. **(a)** Overview of the combination approach to drug repositioning. **(b)** The NeMoCAD gene network analysis tool is a drug repurposing algorithm that uses Bayesian statistical network analysis combined with data from publicly available datasets (e.g., LINCS, KEGG, CTD, TRRUST) for reference transcriptional signatures and to define regulatory network architecture. The algorithm identifies transcriptome-wide differential expression profiles between two biological states (e.g., healthy vs. diseased) in experimental or published transcriptomic datasets and defines the target normalization signature, i.e., the subset of genes that would need to reverse their expression to revert one state to the other. The output of NeMoCAD includes correlation and causation predictions for numerous chemical compounds and approved drugs in the LINCS database based on their ability to reverse the differential expression profile of interest. **(c)** Patient selection and analysis of electronic health records. (Figure panels a & b created with BioRender.com.).

Throughout the pandemic, multiple scientific teams predicted that existing drugs could be repurposed as potential COVID-19 therapeutics computationally through the application of high-throughput *in silico* screens based on artificial intelligence, network diffusion, or network proximity algorithms using the human interactome, SARS-CoV-2 targets, drug targets, docking structures, or biomedical literature as algorithmic inputs [1–3]. These screens proposed hundreds of potential therapeutic options and led to further testing in SARS-CoV-2-infected culture systems and animal models [1]. However, while *in vitro* and pre-clinical testing have offered promising predictions, clinical validation and translation of predicted compounds are much more challenging and few, if any, of these drugs proposed to be repurposed for COVID-19 have demonstrated clinical efficacy. Thus, there is a need for combining improved drug prediction capabilities, despite complex and often inadequately understood biology, with real world evidence, such as electronic health records (EHRs) [4,14], to better inform which predicted compounds should advance toward clinical evaluation (Fig 1A). A related approach has previously been used to identify repurposed drugs for coronary artery disease by integration of protein-protein interaction network proximity and large-scale patient-level longitudinal data [15].

With drug repurposing in mind, we used a Network Model for Causality-Aware Discovery (NeMoCAD) computational tool based on Bayesian statistical network modeling [16] to analyze transcriptomics signatures in tissue samples obtained from COVID-19 positive patients or SARS-CoV-2 infected human cell or organoid cultures to identify FDA-approved drugs that shift the host transcriptomic response to SARS-CoV-2 towards a healthy state (Fig 1B). This approach was agnostic as it was accomplished without an *a priori* defined drug target or mechanism of action.

This computational analysis revealed that a subset of commonly administered statins was among the drugs most frequently predicted to revert the genome-wide gene expression profile of COVID-19 samples to that of a healthy state. Despite limited chemical diversity, statins induce a range of side effects that differ between individual statins [17,18] suggesting the potential for distinct biological activities outside their known shared HMGCR mechanism, which potentially could be harnessed for drug repurposing and eventual new drug development. To explore this possibility in a clinical setting, a retrospective analysis was carried out using a database containing EHRs of over 490,000 COVID-19 patients, more than 4,000 of which are from patients who are actively taking statins (Fig 1C). This analysis demonstrated that use of only a subset of statins, including simvastatin and atorvastatin, correlated with decreased morbidity and increased survival in COVID-19 patients, confirming hidden divergent activities within a seemingly homogeneous drug class. Experimental *in vitro* studies confirmed that the drug most frequently predicted to reverse the COVID-19 state and that correlated with decreased morbidity in EHRs–simvastatin–also potently inhibited infection of Vero6 cells by SARS-CoV2 *in vitro*. However, other statins were less effective, suggesting that individual statins might have molecule-specific activities beyond the shared HMGCR target and thus vary in their ability to protect COVID-19 patients.

## Methods

### Ethics statement

The EHR study was approved by the University of California, San Francisco, institutional review board.

### Transcriptomic-based compound prediction for drug repurposing

The drug prediction software, NeMoCAD (Network Modeling for Causal Discovery), was used to predict compounds that would mimic the shift from a COVID-19-positive state to a control state [16]. NeMoCAD is a drug repurposing algorithm that performs correlation analysis of transcriptional gene signatures and a Bayesian statistical analysis of a network comprised of drug-gene and drug-drug interactions to identify compounds capable of changing a transcriptional signature indicative of disease to a healthy state (e.g., reverting a disease state to a healthy state) [16]. First, transcriptome-wide differential expression profiles are identified between two biological states in the input transcriptomic dataset (experimental or published) and this profile is used to define the target normalization signature (Fig 1B). We define this target signature as the subset of genes whose expression levels need to be reversed in order to revert one state to the other (e.g., normalize the diseased state). Then, pairwise analysis is carried out based on the comparison between gene expression profiles of compounds found in the LINCS database release v1 and the target normalization signature (Fig 1B). NeMoCAD computes multiple correlation statistics (e.g., Pearson correlation, cross entropy) across all differentially expressed genes as well as a combination score (e.g., Pearson correlation divided by the cross-entropy). The pairwise analyses implicitly assume genes are expressed independently of one another.

NeMoCAD then separately generates putative predictions that incorporate gene-gene dependencies based on Bayesian network analysis on a regulatory and drug-gene interaction network architecture defined using publicly available databases of gene-gene interactions based on single gene knockout datasets in human cells (KEGG, TRRUST) [19–21] and reference transcriptional signatures of drugs (LINCS, CTD) (Fig 1B) [22,23]. NeMoCAD parses the perturbation signatures from the LINCS dataset, and estimates the conditional probability of upregulation of all LINCS genes given a certain drug is present (drug-gene probability). Drug-gene probabilities are averaged over the multiple treatment time points (6 and 24 hours) provided for each drug in LINCS.

NeMoCAD combines a directed unweighted network structure with interaction probabilities between drugs and genes for the connecting network edges. Together, these features generate a weighted directed graph consisting of all possible paths that connect at least 2 genes of interest from the relevant genes within the target transcriptomic normalization set and the drugs. This approach encodes the entire region of influence of a given list of genes and the drugs that can reverse their gene expression profiles in the desired manner. The network generated from database and transcriptomic information is then used as input for a message-passing algorithm (e.g., loopy belief propagation algorithm). The marginal probability distributions of drugs being “on” given the expression state of every gene are computed using the joint probability distribution. Drugs with a low probability of being “on” (probability < 0.5) are filtered out from prediction lists. Drugs are then ranked based on the combination score derived from the correlation metrics (Pearson correlation and cross entropy). As a robustness criterion, drug predictions were assessed at multiple fold change thresholds to identify drugs that are consistently associated with the subnetwork defined by the differential expression profile.

Using 14 publicly available transcriptomic datasets derived from human patients, tissue samples, organoids, and cells (Table 1) [24–30], NeMoCAD identified transcriptome-wide differential expression profiles between the control and COVID-19 states for each dataset and defined a target normalization signature to mimic, which would shift the transcriptome from a COVID-19 disease to control state. The frequency of NeMoCAD predictions across input datasets was evaluated for each statin within the LINCS database and statins with LINCS gene expression probabilistic network profiles that most strongly correlate with the target normalization signature were identified. We assessed the frequency of predictions within the top 25% of drugs, ranked by combination score for each dataset. We selected the 25% threshold based on our prior experience using the NeMoCAD platform, during which we have typically selected predictions within the top quartile for further in vitro or in vivo screening. Prediction frequency was also stratified based on tissue source and type (Table 1). To understand differences in the reference transcriptional data that could influence drug predictions, we compared the LINCS drug-gene probability signatures for each statin by principal component analysis (PCA). Using the time-averaged drug-gene probabilities from LINCS for 12,328 genes across 7 statins of interest (atorvastatin, fluvastatin, lovastatin, pitavastatin, pravastatin, rosuvastatin, and simvastatin), we created a PCA object in the R package ggfortify using the autoplot function. The package ggplot2 was used for plotting customizations. R version 4.0.5 was used for all computations and plotting.

**Table 1:** RNA-sequencing datasets used as inputs for network-based drug predictions.

Dataset Source	Public Identifier/Link	Sample Source	Tissue/Cell Type	Conditions assessed for predictions	COVID-19 samples	Control samples	Drugs predicted
**Greninger** ^**17**^	GSE152075	Nasal swab samples from COVID patients	Nasopharyngeal	COVID > healthy	430	54	377
**Mertz** ^**18**^	GSE151764	Autopsy samples from COVID patients	Lung	COVID > healthy	16	6	623
**Mertz** ^**18**^	GSE151764	Autopsy samples from COVID patients	Lung	COVID (patients w/ normal BMI) > healthy	2	6	647
**Mertz** ^**18**^	GSE151764	Autopsy samples from COVID patients	Lung	COVID (patients w/ high BMI) > healthy	14	6	571
**OSF**	https://osf.io/7nrd3/	COVID patients	Blood PBMC	COVID > healthy	3	3	330
**OSF**	https://osf.io/7nrd3/	COVID patients	Bronchoalveolar lavage fluid	COVID > healthy	32	54	436
**Redmond** ^**16**^	GSE151803	Organoids	Liver	SARS-CoV-2 infected > mock treated	6	6	810
**Redmond** ^**16**^	GSE151803	Organoids	Pancreas	SARS-CoV-2 infected > mock treated	3	3	393
**Svendsen** ^**19**^	GSE150392	Human induced pluripotent stem cell-derived cells	Cardiomyocytes	SARS-CoV-2 infected > mock treated	3	3	716
**Takayama** ^**14**^	GSE150819	Organoids	Bronchial epithelial cells	SARS-CoV-2 infected > mock treated	3	3	719
**tenOever** ^**20**^	GSE147507	Cultured cells infected with SARS-CoV-2	A549	SARS-CoV-2 infected > mock treated	3	3	480
**tenOever** ^**20**^	GSE147507	Autopsy samples COVID patients	Lung	COVID > healthy	2	2	576
**tenOever** ^**20**^	GSE147507 & GSE200074	Autopsy samples COVID patients	Lung	COVID > healthy	2	2	711
**Ting** ^**15**^	GSE150316	Autopsy samples from COVID patients	Lung	COVID > healthy	52	5	384

### Electronic health record analyses

Data from the Cerner Real World Data COVID-19 deidentified EHR database containing records of 490,373 patients with a diagnosis of COVID-19 or COVID-19 exposure across 87 health care centers were analyzed. The following statins were included: atorvastatin, fluvastatin, lovastatin, pitavastatin, pravastatin, rosuvastatin, and simvastatin. Primary outcome was death after the onset of COVID-19. Inclusion criteria, considered comorbidities, and statistical analysis are detailed in the Methods in S1 Text.

### Viral infection of Vero6 cells with SARS-CoV-2 virus

All work with native SARS-CoV-2 virus was performed in a BSL3 laboratory and approved by our Institutional Biosafety Committee. All drug screens to assess SARS-CoV-2 inhibition and cytotoxicity were performed with Vero E6 (Vero6) cells (ATCC# CRL 1586) using published methods (Methods in S1 Text) [31]. A curve fitting procedure was used to determine IC50 and CC50 values (Methods in S1 Text).

### Viral infection and host response of HUVECs with the OC43 virus

To measure the impact of selected drugs on HCoV-OC43 infection, 96-well plates seeded with human umbilical vein endothelial cells (HUVECs) were infected with HCoV-OC43 and treated with drugs (Methods in S1 Text). Viral load, Hoechst fluorescence, and IP-10 measurements were measured and normalized to vehicle control samples for each assay. Each group was compared to vehicle controls using the Brown-Forsythe and Welch ANOVA tests and corrected for multiple comparisons using a Dunnett T3 test.

### Visualizations

Plotting was performed in Prism 9 (GraphPad Software LLC) or in R versions 3.0.2 and 4.0.5. Schematic in Fig 1 was made in Biorender.

### Role of the funding source

Funding sources were not involved in study design, in the collection, analysis, and interpretation of data, or in the writing of the report.

## Results

**Fig 2:**
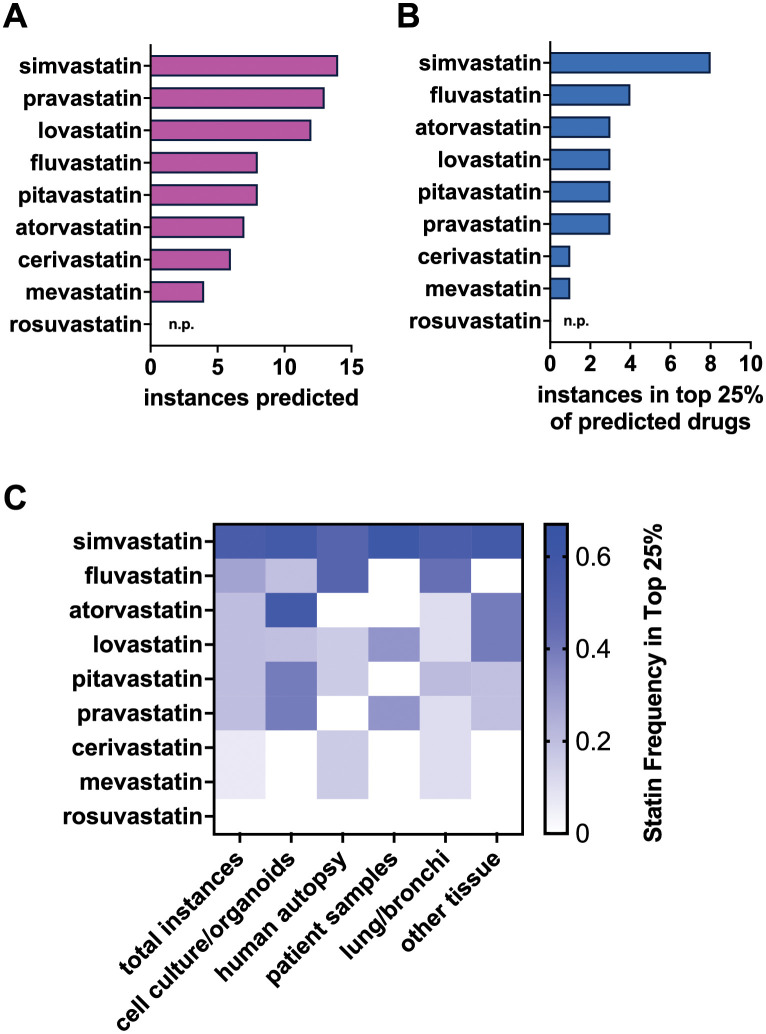
Simvastatin is identified by gene network-based predictions as the most likely drug in its category to reverse COVID-19 transcriptional profiles. **(a)** Statins are predicted to shift the COVID-19 state to a healthy state, with simvastatin predicted for all datasets analyzed (14). Rosuvastatin was the only statin not predicted (n.p.) for any COVID-19 transcriptomics signatures. **(b)** 8 of 9 statins in the LINCS database were in the top 25% of drugs predicted for at least one dataset investigated. **(c)** Frequency of prediction for each statin when input datasets are stratified by sample source and tissue origin. Prediction frequency is normalized by the number of input datasets from each sample source and tissue origin.

The NeMoCAD gene network analysis tool [16] was used to identify FDA-approved drugs predicted to normalize the COVID-19 gene expression profile based on transcriptomic signatures of human cells or organoids infected with SARS-CoV-2 as well as cells or tissues obtained from COVID-19 patients or healthy control subjects. NeMoCAD identified gene changes across the transcriptome, compared them with gene expression changes induced by approved drugs in existing databases (e.g., LINCS, KEGG, TRRUST, CTD), and then prioritized compounds based on their ability to shift the disease transcriptomic signature state back to a healthy state (Fig 1A). COVID-19 normalizing drugs were predicted based on 14 differential RNA-seq expression datasets (COVID-19 vs. healthy) from 12 independent transcriptomics studies (Table 1). Across all datasets, NeMoCAD prioritized a different number of drugs for each dataset (Table 1), with 172 drugs representing the intersection of all these results and therefore shared drugs relevant to all samples (Table 2). Of the 2,436 drugs we investigated across the 14 differential expression datasets, 1,477 of the 2,436 drugs were not predicted to normalize any disease signature. Across the 959 compounds predicted to normalize at least one disease signature, each drug was predicted on average by 8.1 of the 14 datasets. Surprisingly, we found multiple statins that inhibit HMGCR to be predicted more frequently than expected by the average, with simvastatin predicted 14/14 times, pravastatin 13/14 times, and lovastatin 12/14 times (Fig 2A). Of the 9 statins included in the NIH LINCS program database, 8 were in the top 25% of drugs predicted for at least one dataset investigated (Fig 2B). Across all datasets, simvastatin and fluvastatin were most frequently among the top 25% of predicted compounds.

**Table 2:** Drugs predicted 14 times out of the 14 state changes investigated.

3,3’-diindolylmethane	ciglitazone	fenofibrate	mifepristone	sirolimus
4-hydroxy-2-nonenal	cisplatin	fenretinide	morin	sorafenib
acetaldehyde	clodronic-acid	fluorouracil	naringenin	sulfasalazine
acetylcysteine	clofibrate	fluoxetine	niclosamide	sulindac
aflatoxin-b1	clozapine	flutamide	nicotine	tacrolimus
AICA-ribonucleotide	colchicine	folic-acid	nimesulide	tamibarotene
alitretinoin	colforsin	fulvestrant	norepinephrine	tamoxifen
alpha-tocopherol	corticosterone	fumonisin-b1	ochratoxin-a	testosterone
amiodarone	coumestrol	furan	olanzapine	tetrachloroethylene
apigenin	curcumin	gefitinib	omeprazole	tetracycline
artesunate	cycloheximide	gemcitabine	orphenadrine	thalidomide
ascorbic-acid	cyclophosphamide	genistein	oxidopamine	thapsigargin
aspirin	cytarabine	glafenine	paclitaxel	theophylline
azacitidine	dactinomycin	glucosamine	panobinostat	topotecan
azathioprine	dasatinib	haloperidol	pentachlorophenol	tretinoin
belinostat	daunorubicin	hydralazine	phenethyl-isothiocyanate	tributyltin
benazepril	decitabine	ibuprofen	phenytoin	trichloroethylene
benzo(a)pyrene	deguelin	ifosfamide	pifithrin	trichostatin-a
bezafibrate	dexamethasone	indole-3-carbinol	pilocarpine	triclosan
bisphenol-a	dichloroacetic-acid	ionomycin	pirinixic-acid	troglitazone
bortezomib	diclofenac	irinotecan	piroxicam	tunicamycin
bucladesine	dieldrin	isotretinoin	progesterone	tyrphostin-AG-1478
bufalin	diethylstilbestrol	leflunomide	propylthiouracil	urethane
buspirone	dimethylnitrosamine	levofloxacin	pterostilbene	valdecoxib
buthionine-sulfoximine	dinoprost	lithium-chloride	pyrazolanthrone	valproic-acid
cadmium-chloride	disulfiram	losartan	quercetin	vancomycin
caffeine	doxorubicin	luteolin	ranitidine	verapamil
calcitriol	ellagic-acid	melatonin	reserpine	vincristine
camptothecin	emodin	mercaptopurine	resveratrol	vorinostat
capsaicin	entinostat	metformin	rimonabant	wortmannin
carbamazepine	estradiol	methapyrilene	ritonavir	zearalenone
carbon-tetrachloride	estriol	methimazole	rosiglitazone	zidovudine
catechin	ethinyl-estradiol	methotrexate	rotenone	
celecoxib	etoposide	methoxychlor	sertraline	
chlorpromazine	famotidine	mevalonic-acid	simvastatin	

We further assessed our predictions to understand how different types of input data might impact the types of compounds predicted. Stratification of the input datasets by sample source (COVID patient, autopsy sample, or cell culture/organoid) and tissue origin (lung/bronchi or other) revealed that simvastatin is frequently predicted across all dataset types (Fig 2C). In addition, atorvastatin is often predicted when cell culture and organoid samples are used as data inputs, whereas fluvastatin is commonly predicted in human autopsy samples, and lovastatin and pravastatin are predicted at an intermediate frequency using patient input data. Specific investigation of tissue origin revealed that simvastatin and fluvastatin are most frequently predicted when input datasets are derived from lung or bronchi tissue (Fig 2C). Simvastatin, atorvastatin, and lovastatin are also frequently predicted using samples from other non-lung tissues, including nasopharyngeal swabs, blood, liver, pancreas, and cardiac cells.

This finding that statins might differ in their ability to suppress responses to SARS-CoV-2 infection and that these effects could be independent of their common lipid lowering activity induced us to explore whether different statins also exhibit disparate activities in COVID-19 patients. We used the Cerner Real World Data COVID-19 deidentified EHR database to assess the effects of various statins on the survival of COVID-19 patients who were prescribed these medications. This large database represents a diverse population of patients diagnosed with COVID-19 from January to September 2020 with a duration of follow-up of as long as 8 months in 87 health centers across the US. Among 70,308 eligible patients, we identified 4,330 patients with who were prescribed atorvastatin, lovastatin, pravastatin, rosuvastatin, or simvastatin (Fig 1C). There were no patients who were prescribed fluvastatin in this database. The remaining 65,978 patients had no history of statin exposure (control patients). Cohort characteristics are shown in Tables 3 and 4. As disease severity could vary between the medication exposed and unexposed groups, we accounted for the type of encounter (Urgent care, ER, Admission for Observation, or Inpatient) at the time of COVID diagnosis and found that after matching, there was adequate balance in the encounter type between the compared medication exposed and unexposed groups, with the absolute value of standardized mean difference (SMD) of less than 0.1 for encounter type (S1 and S2 Figs in S1 Text). Overall, the propensity score distributions and SMDs of all matched covariates between treated and control groups before and after matching showed adequate balance between groups after matching, with absolute SMD values of less than 0.1 for all covariates, including demographics, heart diseases and other COVID-19 comorbidities, as well as conditions for which statins are prescribed (S1 and S2 Figs in S1 Text).

**Table 3:** Cohort characteristics before propensity score matching (PSM), reflecting differing percentages of characteristics (demographics) with standardized mean differences (SMD) for those prescribed a specific statin compared to the control cohort not treated with a statin.

Characteristic	No Statins	Atorvastatin	SMD	Lovastatin	SMD	Pitavastatin	SMD	Pravastatin	SMD	Rosuvastatin	SMD	Simvastatin	SMD
N	65978	2676		70		13		383		404		784	
Age (mean (SD))	47.3 (18.5)	67.6 (13.4)	-1.26	70.9 (11.5)	-1.54	69.3 (14.6)	-1.32	70.3 (11.9)	-1.48	67.4 (12.4)	-1.28	70.4 (13.1)	-1.44
Age (%)													
18–39	25902 (39.3)	64 (2.4)	1.02	0 (0.0)	1.14	1 (7.7)	-0.80	5 (1.3)	1.07	7 (1.7)	1.05	15 (1.9)	1.04
40–49	11601 (17.6)	180 (6.7)	-0.34	4 (5.7)	-0.38	0 (0.0)	0.65	15 (3.9)	-0.45	31 (7.7)	-0.30	47 (6.0)	-0.37
50–59	11289 (17.1)	490 (18.3)	-0.03	8 (11.4)	0.16	2 (15.4)	0.05	48 (12.5)	0.13	63 (15.6)	0.04	90 (11.5)	0.16
60–69	7987 (12.1)	700 (26.2)	-0.36	19 (27.1)	-0.39	4 (30.8)	-0.47	97 (25.3)	-0.34	110 (27.2)	-0.39	189 (24.1)	-0.32
70–79	4948 (7.5)	658 (24.6)	0.48	21 (30.0)	0.60	0 (0.0)	0.40	129 (33.7)	0.68	129 (31.9)	0.65	222 (28.3)	0.56
80+	4251 (6.4)	584 (21.8)	0.45	18 (25.7)	0.54	6 (46.2)	1.01	89 (23.2)	0.49	64 (15.8)	0.30	221 (28.2)	0.60
Sex (%)													
Female	34842 (52.8)	1284 (48.0)	0.10	36 (51.4)	0.03	7 (53.8)	-0.02	175 (45.7)	0.14	191 (47.3)	0.11	359 (45.8)	0.14
Male	31136 (47.2)	1392 (52.0)	-0.10	34 (48.6)	-0.03	6 (46.2)	0.02	208 (54.3)	-0.14	213 (52.7)	-0.11	425 (54.2)	-0.14
Race (%)													
American Indian or Alaska Native	1425 (2.2)	47 (1.8)	0.03	0 (0.0)	0.21	0 (0.0)	0.21	5 (1.3)	0.07	5 (1.2)	0.07	11 (1.4)	0.06
Asian or Pacific Islander	1461 (2.2)	77 (2.9)	-0.04	5 (7.1)	-0.24	0 (0.0)	0.21	11 (2.9)	-0.04	9 (2.2)	0.00	34 (4.3)	-0.12
Black or African American	12631 (19.1)	432 (16.1)	0.08	13 (18.6)	0.02	2 (15.4)	0.10	102 (26.6)	-0.18	56 (13.9)	0.14	131 (16.7)	0.06
Mixed Racial Group	270 (0.4)	9 (0.3)	0.01	2 (2.9)	-0.19	0 (0.0)	0.09	1 (0.3)	0.03	0 (0.0)	0.09	1 (0.1)	0.05
Other Racial Group	9378 (14.2)	271 (10.1)	0.13	7 (10.0)	0.13	0 (0.0)	0.58	27 (7.0)	-0.23	26 (6.4)	-0.26	74 (9.4)	-0.15
White	40813 (61.9)	1840 (68.8)	-0.15	43 (61.4)	0.01	11 (84.6)	-0.53	237 (61.9)	<0.001	308 (76.2)	-0.32	533 (68.0)	-0.13
Ethnicity (%)													
Hispanic or Latino	30158 (45.7)	1086 (40.6)	0.10	18 (25.7)	0.43	5 (38.5)	0.15	105 (27.4)	0.39	174 (43.1)	0.05	297 (37.9)	0.16
Not Hispanic or Latino	35820 (54.3)	1590 (59.4)	-0.10	52 (74.3)	-0.43	8 (61.5)	-0.15	278 (72.6)	-0.39	230 (56.9)	-0.05	487 (62.1)	-0.16
Encounter Type (%)													
Admitted for Observation	2272 (3.4)	176 (6.6)	-0.14	4 (5.7)	-0.11	0 (0.0)	0.27	24 (6.3)	-0.13	28 (6.9)	-0.16	37 (4.7)	-0.07
Emergency	24603 (37.3)	368 (13.8)	1.03	12 (17.1)	0.92	2 (15.4)	0.98	53 (13.8)	1.03	67 (16.6)	0.94	90 (11.5)	-1.07
Inpatient	38087 (57.7)	2127 (79.5)	-0.95	54 (77.1)	-0.88	11 (84.6)	-1.11	305 (79.6)	-0.95	304 (75.2)	-0.83	655 (83.5)	1.11
Urgent care encounter	1016 (1.5)	5 (0.2)	0.15	0 (0.0)	0.18	0 (0.0)	0.18	1 (0.3)	0.14	5 (1.2)	0.03	2 (0.3)	0.14

**Table 4:** Cohort characteristics before propensity score matching (PSM), reflecting differing percentages of characteristics (conditions, and outcome of death) with standardized mean differences (SMD) for those prescribed a specific statin compared to the control cohort not treated with a statin.

Characteristic	No Statins	Atorvastatin	SMD	Lovastatin	SMD	Pitavastatin	SMD	Pravastatin	SMD	Rosuvastatin	SMD	Simvastatin	SMD
N	65978	2676		70		13		383		404		784	
Condition (%)													
Obese	23567 (35.7)	1151 (43.0)	-0.15	24 (34.3)	0.03	2 (15.4)	0.48	157 (41.0)	-0.11	170 (42.1)	-0.13	298 (38.0)	-0.05
Cancer	2664 (4.0)	188 (7.0)	-0.13	3 (4.3)	-0.01	1 (7.7)	-0.16	30 (7.8)	-0.16	25 (6.2)	-0.10	49 (6.2)	-0.10
Cerebrovascular Disease	2311 (3.5)	378 (14.1)	0.38	8 (11.4)	0.31	2 (15.4)	0.42	51 (13.3)	0.36	60 (14.9)	0.40	91 (11.6)	0.31
Chronic Kidney Disease (CKD)	4650 (7.0)	654 (24.4)	0.49	10 (14.3)	0.24	4 (30.8)	0.64	121 (31.6)	0.65	86 (21.3)	0.42	178 (22.7)	0.45
COPD	3460 (5.2)	386 (14.4)	0.31	7 (10.0)	0.18	2 (15.4)	0.34	60 (15.7)	0.35	40 (9.9)	-0.18	113 (14.4)	0.31
Diabetes	13677 (20.7)	1586 (59.3)	-0.86	44 (62.9)	-0.95	8 (61.5)	-0.91	246 (64.2)	-0.98	232 (57.4)	-0.81	464 (59.2)	-0.85
Heart Diseases	7141 (10.8)	1029 (38.5)	-0.68	22 (31.4)	-0.52	4 (30.8)	-0.51	154 (40.2)	-0.72	164 (40.6)	-0.73	280 (35.7)	-0.62
Hypertension	23146 (35.1)	2206 (82.4)	-1.10	57 (81.4)	-1.07	11 (84.6)	-1.17	339 (88.5)	-1.32	337 (83.4)	-1.13	649 (82.8)	-1.11
High Cholesterol	9302 (14.1)	1932 (72.2)	-1.45	57 (81.4)	-1.83	11 (84.6)	-1.99	304 (79.4)	-1.73	312 (77.2)	-1.64	592 (75.5)	-1.57
Outcome (%)													
Mortality	4235 (6.4)	431 (16.1)	0.31	15 (21.4)	0.44	4 (30.8)	0.66	71 (18.5)	0.37	53 (13.1)	0.23	153 (19.5)	0.40
Healthcare Center Type (%)													
Academic	5689 (8.6)	254 (9.5)	-0.03	4 (5.7)	0.11	0 (0.0)	0.43	46 (12.0)	0.11	30 (7.4)	0.04	60 (7.7)	0.04
Children	263 (0.4)	0 (0.0)	0.09	0 (0.0)	0.09	0 (0.0)	0.09	0 (0.0)	0.09	0 (0.0)	0.09	0 (0.0)	0.09
Community Healthcare	161 (0.2)	6 (0.2)	0.00	0 (0.0)	0.07	0 (0.0)	0.07	0 (0.0)	0.07	0 (0.0)	0.07	1 (0.1)	0.03
Community Hospital	401 (0.6)	13 (0.5)	0.02	0 (0.0)	0.11	0 (0.0)	0.11	6 (1.6)	-0.09	3 (0.7)	-0.02	11 (1.4)	-0.08
Critical Access	39 (0.1)	0 (0.0)	0.03	0 (0.0)	0.03	0 (0.0)	0.03	0 (0.0)	0.03	0 (0.0)	0.03	0 (0.0)	0.03
IDN	51485 (78.0)	2108 (78.8)	-0.02	56 (80.0)	-0.05	10 (76.9)	0.03	280 (73.1)	0.12	309 (76.5)	0.04	607 (77.4)	0.02
Regional Hospital	7940 (12.0)	295 (11.0)	0.03	10 (14.3)	-0.07	3 (23.1)	-0.29	51 (13.3)	-0.04	62 (15.3)	-0.10	105 (13.4)	-0.04
Region (%)													
Northeast	14216 (21.5)	656 (24.5)	-0.07	11 (15.7)	0.15	2 (15.4)	0.16	61 (15.9)	0.14	58 (14.4)	0.19	162 (20.7)	0.02
Midwest	5196 (7.9)	207 (7.7)	0.01	3 (4.3)	0.15	0 (0.0)	0.41	29 (7.6)	0.01	24 (5.9)	0.08	53 (6.8)	0.04
South	26143 (39.6)	1012 (37.8)	0.04	23 (32.9)	0.14	9 (69.2)	-0.62	194 (50.7)	-0.22	227 (56.2)	-0.34	289 (36.9)	0.06
West	20423 (31.0)	801 (29.9)	0.02	33 (47.1)	-0.34	2 (15.4)	0.38	99 (25.8)	0.11	95 (23.5)	0.17	280 (35.7)	-0.10

Importantly, among individual statins, we found that only treatment with atorvastatin, rosuvastatin, or simvastatin was associated with a statistically significant decrease in the relative risk of death in statin-treated patients compared to matched controls (Table 5). The mortality rate among atorvastatin-treated patients was 16.1% (431 of 2676) versus 20.4% (545 of 2676) among matched untreated control patients, with a reduction of 14% in the RR (0.86 [95% CI, 0.83–0.93]; Bonferroni adjusted p-value = 6.24E-05) (Table 5 and S1 Table in S1 Text). The mortality rate among rosuvastatin-treated patients was 13.1% (53 of 404) and 21.0% (850 of 4040) among matched untreated control patients, with a reduction of 41% in the RR (0.59 [95% CI, 0.45–0.78]; adjusted p-value = 9.61E-05) (Table 5 and S4 Table in S1 Text). The mortality rate among simvastatin-treated patients was 19.5% (153 of 784) and 23.3% (914 of 3920) among matched untreated control patients, with a reduction of 17% in the RR (0.83 [95% CI, 0.70–0.97]; adjusted p-value = 0.02) (Table 5 and S5 Table in S1 Text).

**Table 5:** Mortality rates of patients treated with (A) atorvastatin, (B) lovastatin, (C) pravastatin, (D) rosuvastatin, and (E) simvastatin, and matched control groups, and relative risk of death with 95% confidence interval and Benjamini-Hochberg adjusted p-value from the iteration with the least significant result for each comparison.

	treated patients		controls			
Statin (moderate dose)	Mortality rate, %	No. died/No. treated	Mortality rate, %	No. died/No. treated	Relative risk (95% CI)	Adjusted P-value*
Atorvastatin	16.1	431/2676	20.4	545/2676	**0.86 (0.80–0.93**)	6.24E-05
Lovastatin	21.4	15/70	19.1	134/700	1.14 (0.66–1.96)	0.76
Pravastatin	18.5	71/383	23.1	883/3830	0.78 (0.61–1.00)	0.05
Rosuvastatin	13.1	53/404	21.0	850/4040	**0.59 (0.45–0.78)**	9.61E-05
Simvastatin	19.5	153/784	23.3	914/3920	**0.83 (0.70–0.97)**	0.02

**Fig 3:**
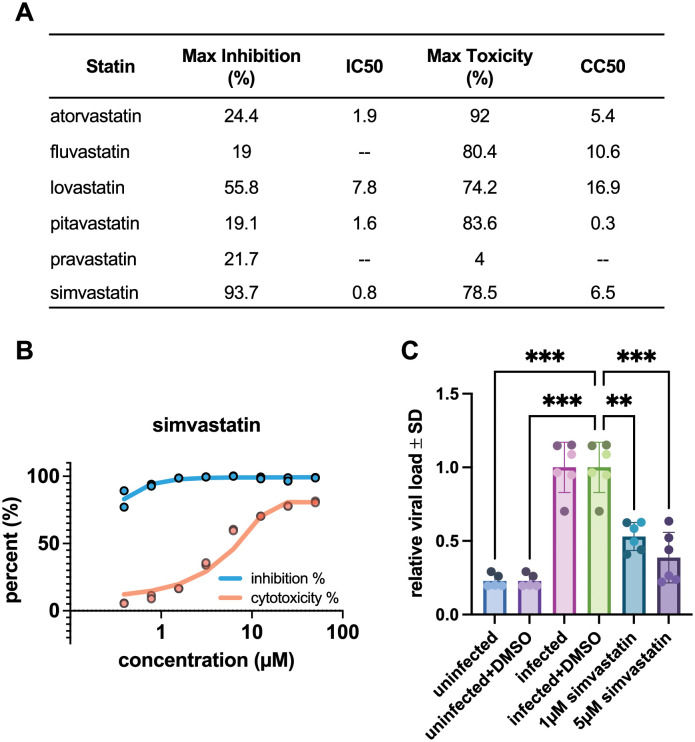
Effects of statins on coronavirus infections *in vitro*. **(a)** Inhibitory and cytotoxicity parameters from SARS-CoV-2 infection of Vero6 wild-type or GFP-expressing cells for a subset of statins contained in the LINCS database. Mean parameters for each statin are derived from two independent experiments. **(b)** Dose-response curves demonstrating the ability for simvastatin to inhibit GFP-SARS-CoV-2 infection (MOI = 0.1) in a dose-dependent manner in Vero6 cells. **(c)** Simvastatin also inhibits the human coronavirus, OC43, in HUVEC cells when added at 1 or 5 μM concentrations (***p < 0.001, **p < 0.01). Error bars represent average ± SD; repeated in n = 2 independent biological experiments displayed in light (experiment 1) and dark (experiment 2) shaded data points.

Statins were also tested as part of a larger drug screening program in SARS-CoV-2-infected Vero6 cells. Within the statin drug class, simvastatin most potently inhibited infection with a half maximal inhibitory concentration (IC_50_) of 0.8 μM and almost a 10-fold higher 50% cytotoxic concentration (CC_50_ = 6.5 μM) (Fig 3A and 3B). The other statins were either unable to significantly inhibit SARS-CoV-2 infection in Vero6 cells or they were found to be toxic at doses required to see inhibitory effects (Fig 3A and S3 Fig in S1 Text). It is important to note that discordance observed between predictions made by NeMoCAD and SARS-CoV-2 inhibition in Vero6 cells is expected since NeMoCAD predicts drugs that will affect host response to infection, and not necessarily directly act on the virus to inhibit infection (e.g., reduce entry or replication). However, we also know that viral replication induces a host response and the transcriptional outcome of infection will always depend on interactions occurring within the virus-host system [32,33]. Recent studies examining multiple coronavirus infections suggest that coronavirus infection signatures can be reversed in the host using known anti-SARS-CoV-2 inhibitors and that regulating cholesterol homeostasis and microtubule cytoskeleton organization in the host might contribute to antiviral efficacy [34,35]. Therefore, we cannot completely decouple the effects of predicted drugs on viral inhibition versus more conventional host measurements, such as cytokine levels. Furthermore, we found that simvastatin also inhibited infection of HUVECs by a related coronavirus (OC43) (Fig 3C) and potently reduced cytokine (IP-10) production without cytotoxic effects (S4 Fig in S1 Text). Similar results were observed with IL-6 and GM-CSF although the virus induced levels of these cytokines were variable. Thus, this particular statin appears to exhibit direct antiviral activity in addition to the HMGCR activity it shares with the other stains.

## Discussion

Taken together, these data show that *in silico* prediction based on transcriptomics datasets from human patients, tissues, and cells combined with clinical database analyses offer a useful approach for identifying and validating non-obvious effects of FDA-approved drugs, thereby enabling rapid repurposing of these compounds. With multiple lines of evidence coming together, we found that clinical observations cannot always be explained by experimental biological studies, and vice versa. However, we demonstrate here that by looking at multiple lines of evidence in parallel, drug repurposing candidates are derisked across multiple biological levels to enable actionable repurposing interventions and potential new target discovery efforts.

Past retrospective studies suggested that patients prescribed drugs within the statin class have an overall lower risk of mortality from COVID-19 [4,6,8], although this was not observed in one study [10]. Predictions by NeMoCAD suggested that statins may differ in their ability to induce a shift from COVID-19 to healthy states, which could in part explain differences in results between these studies if the statin types differed [4–6,10]. Specifically, NeMoCAD predicted that simvastatin, fluvastatin, and atorvastatin were the most likely statins to normalize the COVID-19 gene expression profile. Indeed, when we analyzed mortality in a large EHR database of patients with COVID-19, we confirmed that there are differences in the mortality risks of COVID-19 patients prescribed the different statins compared to their respective matched control cohort, with simvastatin, atorvastatin, and rosuvastatin associated with a significant reduction in the relative risk of death. We were unable to find a statistically significant difference in mortality risk among patients prescribed lovastatin or pravastatin represented in our EHR database compared to their respective matched control cohorts. Unfortunately, there were no patients who took fluvastatin and only 13 who took pravastatin in our EHR database, so we cannot make any conclusions about the protective effects of these compounds. Exploring an EHR database with a greater number of patients prescribed these statins in the future should allow for greater insights into any differences in mortality risk associated with these particular drugs.

Simvastatin and atorvastatin were predicted to be active by NeMoCAD, while rosuvastatin was not. Since the *in silico* predictions were based on the *in vitro* LINCS database, we expected some differences to be present between the predictions and EHR outcomes. NeMoCAD’s transcriptomics-based predictions do not take into account the physical properties (S6 Table and S5 Fig in S1 Text) or potential antiviral activity for a drug. Structurally, rosuvastatin has a pyrimidine side ring, which makes it unique from the other statins which have pyrrole, naphthalene, or other side ring structures [36]. Interestingly, remdesivir and molnupiravir, two of the few drugs to date that have received emergency use authorization (EUA) by the FDA for treatment of COVID-19 [37,38], are nucleoside analogues—chemically synthesized analogs of pyrimidines and purines [39]. Remdesivir, molnupiravir, and other nucleosides have been shown to directly block SARS-CoV-2 infection in vitro and/or in animal models [39–43]. The antiviral effects of nucleoside analogues is believed to result from their incorporation specifically by viral polymerases leading to defects in viral replication or through their antimetabolite activity where they compete with cellular enzymes for their natural ligands [39]. We did not assess rosuvastatin in our in vitro screens of viral inhibition; however, in work by Ahmed et al, their structure-based drug repositioning approach for drugs with potential inhibitory effects on COVID-19 virus predicted anti-viral drugs and rosuvastatin among their top six hits and demonstrated the inhibitory effects on SARS-CoV-2 replication by rosuvastatin and other drugs in VeroE6 cells [44]. The physical properties of rosuvastatin, in particular its structural similarity to nucleoside analogues, may confer this statin with additional antiviral properties not had by the other statins that we assessed, and could explain why our EHR analysis found rosuvastatin as associated with a reduction in the relative risk of death in patients with COVID-19; whereas, NeMoCAD did not predict this drug.

**Fig 4:**
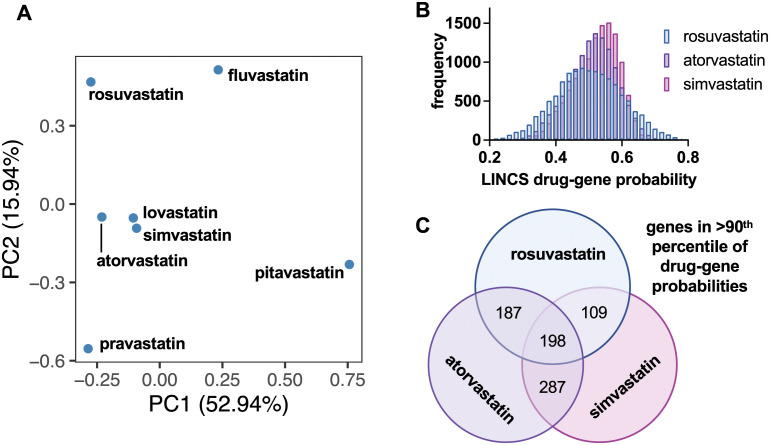
Comparison of LINCS drug-gene interaction probability data for each statin. **(a)** PCA plots of drug-gene interaction data for each statin reveals clustering of simvastatin, atorvastatin, and lovastatin when assessed across all genes in this dataset. **(b)** Distribution of LINCS drug-gene probabilities for statins that reduce mortality in patients. **(c)** Simvastatin, atorvastatin, and rosuvastatin share top gene targets (> 90^th^ percentile), with the most shared between atorvastatin and simvastatin.

Moreover, assessment of the LINCS data that defines the probability of each drug affecting specific genes combined with PCA also revealed closer clustering amongst simvastatin, lovastatin, and atorvastatin, which is consistent with their frequent co-prediction, whereas rosuvastatin is more distant (Fig 4A). Global analysis of the mortality-reducing statins also shows greater similarity in LINCS probability distribution between simvastatin and atorvastatin, whereas a wider probability distribution is observed for rosuvastatin (Fig 4B), and a detailed comparison of the shared top gene targets (> 90^th^ percentile) between these three statins similarly revealed that the most genes are shared between simvastatin and atorvastatin (Fig 4C). Therefore, these two statins may act in a more similar manner than rosuvastatin, which could influence the COVID-19 response through alternative mechanisms (e.g., direct antiviral and/or post-transcriptomic effects) that would not be detected using our transcriptomics-based computational approach. Additionally, various statins are typically dosed based on their cholesterol and lipid lowering properties, not based on their immune modulating properties; thus, a drug that appears to be most effective in silico for one of its "side-effect" properties might not appear to have a similar effect in vivo simply because of dosing decisions in practice. The discordance observed between drug predictions and EHR outcomes suggests that either technique alone is insufficient to identify drugs for clinical use and therefore the combination of methods is essential for narrowing the list of drug candidates before inclusion in randomized trials.

Collectively, our results suggest that statins exhibit divergent effects on the *host response* to SARS-CoV-2 infection despite a shared annotated target and common mechanism for treating dyslipidemia. In addition to their host-modulating effects, earlier work indicates that patients on statins seem to have improved outcomes following bacterial infection, an effect which is especially pronounced with respiratory tract infections, including pneumonia [45]. However, meta-analysis of these studies reveals mixed results, again potentially suggesting that statins do not act uniformly as infection modulators. These protective effects of statins against infection may be due to their well-documented anti-inflammatory and immunomodulatory properties [46,47]. Although originally developed to lower serum cholesterol, accumulating evidence suggests that statins have strong anti-inflammatory effects that contribute to their beneficial effects in patients experiencing vascular disease like atherosclerosis [46]. Furthermore, statins may upregulate HO-1 [7], which is a central modulator of the immune system, affecting anti-inflammation and anti-oxidation, which could prevent the severe “cytokine storm” inflammatory response that is central to morbidity and mortality in COVID-19 patients [48]. By upregulating HO-1, statins, including simvastatin, lovastatin, atorvastatin, or rosuvastatin, also can increase the production of carbon monoxide and bilirubin [9], both of which have immunomodulatory, antioxidative, and anti-inflammatory characteristics. In addition, statins may reduce the likelihood of graft-versus-host disease by inhibiting antigen presentation and shifting pro-inflammatory responses toward anti-inflammatory responses [47]. We do not know precisely why certain statins appear to have superior disease-modifying activity than others. Unlike databases such as DrugBank that outline the known mechanisms of action, NeMoCAD surveys all possible interactions within the transcriptome and thus more work is needed to understand the clinical targets. However, longitudinal studies of the transcriptome in COVID-19 patients prescribed diverse statins could help clarify the mechanisms involved in these responses.

Despite strong evidence of their host modulating properties, there are also preliminary indications that some statins have more direct antibacterial and antiviral capabilities. Our *in vitro* studies with simvastatin suggest that at least this statin type can exhibit direct antiviral activity against both SARS-CoV-2 and the common cold coronavirus OC43, independently of its known lipid-lowering action. Simvastatin has previously been shown to exhibit superior antibacterial effects compared to fluvastatin and pravastatin, including against *S*. *pneumoniae* and *M*. *catarrhalis* infections [45,49]. In the case of bacterial infection, it has been suggested that the activity of simvastatin is linked to its hydrophobicity, which may perturb the bacterial cell membrane compared to the more hydrophilic fluvastatin and pravastatin [49]. The hydrophobicity of statins is also one characteristic that can influence their target-binding characteristics and pharmacokinetic profile, potentially impacting how statins act on a multitude of organ systems and disease states [50]. Critically, the pro-drug form of simvastatin, predicted computationally and tested in our *in vitro* assays, is rapidly metabolized to its active acid form *in vivo* and therefore only a fraction of the total dose is maintained as the original pro-drug. We also observed much weaker inhibition of SARS-CoV-2 infection when treating *in vitro* with the hydroxy acid metabolized form of simvastatin in Vero6 cells relative to treatment with the pro-drug form of simvastatin (S6 Fig in S1 Text). Therefore, future work should investigate simvastatin combined with an inhibitor of cytochrome P450, a hemeprotein that plays a key role in the metabolism of drugs. By limiting drug metabolism, it would be possible to better assess if the simvastatin pro-drug is responsible for anti-viral effects *in vivo*.

NeMoCAD predicted a diverse array of drugs from COVID-19 datasets as it works outside of the very limited "on target mechanisms" reported to the FDA or otherwise well understood. This enables us to capture potential off-target effects that may be present across many drugs; however, the identification of specific pathways and/or genes that are key for the effects of the various drugs predicted by NeMoCAD remains challenging. Many of the potential COVID-19 therapeutics identified using past computational drug repurposing strategies failed when tested either using in vitro culture models, animals, or in the clinic. In fact, very preliminary *in vivo* studies by our team showed no effect of simvastatin on infection nor host response in SARS-CoV-2-infected hamsters and mice, despite attaining plasma levels that were greater than the IC_50_ for inhibition of infection and host response *in vitro*. These contrasting results highlight the challenges involved in conducting translational investigation from *in silico* predictions to *in vitro*, pre-clinical, and clinical studies, in particular raising questions about the clinical translation relevance of any disease model. Leveraging large patient databases early in the drug repurposing process to validate drugs predicted by computational approaches makes it possible to estimate how potential repurposed drugs may perform in infected patients and clinical sub-populations who regularly take these medications for other diseases or disorders. Importantly, in these databases we cannot completely rule out a possibility of unaccounted confounders correlated both with the drug and mortality, nor can we be confident that the medical histories of all patients are represented accurately as some information may be incomplete. Moreover, the retrospective nature of EHR analysis only allows us to identify an association between statin treatment and COVID-19 mortality, but not causal effects. Therefore, we envision that the process of generating *in silico* predictions and validating in databases will be a means to further narrow drug candidates and identify a more curated collection of therapeutics for testing in randomized control trials. Considering the continuing challenges with vaccine distribution and uptake, as well as the vulnerability of older populations to COVID-19, understanding non-obvious effects of approved drugs on patient mortality from infectious disease will be useful for combating this pandemic as well as ones that are likely to emerge in the future.

Finally, our findings suggest that drug repurposing efforts may require consideration of the many molecule-specific effects rather than taking reported drug targets and mechanisms at face value. Indeed, we believe that our approach, which counterpoints computational network-level analysis of biological interactions with *in vitro* exploratory screening and retrospective clinical evidence analysis, may form the basis of an altogether more powerful repurposing strategy in a pandemic scenario.

## Supporting information

S1 Text Methods. Detailed methodology for electronic health record analyses and *in vitro* experiments. **S1 Fig**. Density plots of the distribution of propensity scores for the patients treated with (A) atorvastatin, (B) lovastatin, (C) pravastatin, (D) rosuvastatin, and (E) simvastatin, and control groups before and after matching. **S2 Fig**. Absolute standardized differences in means for matched categorical and continuous variables between patients treated with (A) atorvastatin, (B) lovastatin, (C) pravastatin, (D) rosuvastatin, and (E) simvastatin, and control groups for all data (before matching) and matched data. **S3 Fig**. Dose-response curves demonstrating the effects of statins on SARS-CoV-2 infection (MOI = 0.1) in Vero6 cells. **S4 Fig**. Simvastatin added at 1 or 5 μM concentrations in HUVEC cells infected with human coronavirus, OC43, modulates IP-10 levels without significant cytotoxicity detected by Hoechst fluorescence (***p < 0.001, **p < 0.01, *p < 0.05). Error bars represent average ± SD; repeated in n = 2 independent biological experiments displayed in light (experiment 1) and dark (experiment 2) shaded data points. **S5 Fig**. Chemical structures of different statins. **S6 Fig**. Dose-response curves demonstrating the differential effects of (A) simvastatin and (B) its active acid form, simvastatin hydroxy acid, on SARS-CoV-2 infection (MOI = 0.1) in Vero6 cells. **S1 Table**. Propensity score (PS) matching by demographics, encounter type at the time of the first recorded COVID-19 diagnosis, COVID-19 comorbidities, and prescription indications: mortality rates for atorvastatin-exposed and PS-matched unexposed patients, a relative risk [95% confidence interval] and Benjamini-Hochberg adjusted p-value in each iteration. **S2 Table**. Propensity score (PS) matching by demographics, encounter type at the time of the first recorded COVID-19 diagnosis, COVID-19 comorbidities, and prescription indications: mortality rates for lovastatin-exposed and PS-matched unexposed patients, a relative risk [95% confidence interval] and Benjamini-Hochberg adjusted p-value in each iteration. **S3 Table**. Propensity score (PS) matching by demographics, encounter type at the time of the first recorded COVID-19 diagnosis, COVID-19 comorbidities, and prescription indications: mortality rates for pravastatin-exposed and PS-matched unexposed patients, a relative risk [95% confidence interval] and Benjamini-Hochberg adjusted p-value in each iteration. **S4 Table**. Propensity score (PS) matching by demographics, encounter type at the time of the first recorded COVID-19 diagnosis, COVID-19 comorbidities, and prescription indications: mortality rates for rosuvastatin-exposed and PS-matched unexposed patients, a relative risk [95% confidence interval] and Benjamini-Hochberg adjusted p-value in each iteration. **S5 Table**. Propensity score (PS) matching by demographics, encounter type at the time of the first recorded COVID-19 diagnosis, COVID-19 comorbidities, and prescription indications: mortality rates for simvastatin-exposed and PS-matched unexposed patients, a relative risk [95% confidence interval] and Benjamini-Hochberg adjusted p-value in each iteration. **S6 Table**. Statin properties. Adapted from references [36,51].
(DOCX)

S1 Form NeMoCAD EULA Form. If you are a user from an academic or non-profit research institution, please fill out this form to obtain access to NeMoCAD.
(DOCX)

## References

[1] Gysi DM, do Valle Í, Zitnik M, Ameli A, Gan X, Varol O, et al. Network medicine framework for identifying drug-repurposing opportunities for COVID-19. Proc Natl Acad Sci U S A. 2021;118: 1–11. doi: 10.1073/pnas.2025581118 33906951 PMC8126852

[2] Dae Jang W, Jeon S, Kim S, Yup Lee S. Drugs repurposed for COVID-19 by virtual screening of 6,218 drugs and cell-based assay. Proceedings of the National Academy of Sciences. 2021;118: 1–9. doi: 10.1073/pnas.2024302118/-/DCSupplemental PMC8325362 34234012

[3] Zhou Y, Hou Y, Shen J, Mehra R, Kallianpur A, Culver DA, et al. A network medicine approach to investigation and population-based validation of disease manifestations and drug repurposing for COVID-19. PLoS Biol. 2020;18. doi: 10.1371/journal.pbio.3000970 33156843 PMC7728249

[4] Marić I, Oskotsky T, Kosti I, Le B, Wong RJ, Shaw GM, et al. Decreased Mortality Rate Among COVID-19 Patients Prescribed Statins: Data From Electronic Health Records in the US. Front Med (Lausanne). 2021;8: 1–8. doi: 10.3389/fmed.2021.639804 33614688 PMC7887302

[5] de Spiegeleer A, Bronselaer A, Teo JT, Byttebier G, de Tré G, Belmans L, et al. The Effects of ARBs, ACEis, and Statins on Clinical Outcomes of COVID-19 Infection Among Nursing Home Residents. J Am Med Dir Assoc. 2020;21: 909–914.e2. doi: 10.1016/j.jamda.2020.06.018 32674818 PMC7294267

[6] Zhang XJ, Qin JJ, Cheng X, Shen L, Zhao YC, Yuan Y, et al. In-Hospital Use of Statins Is Associated with a Reduced Risk of Mortality among Individuals with COVID-19. Cell Metab. 2020;32: 176–187.e4. doi: 10.1016/j.cmet.2020.06.015 32592657 PMC7311917

[7] Hsu M, Muchova L, Morioka I, Wong RJ, Schröder H, Stevenson DK. Tissue-specific effects of statins on the expression of heme oxygenase-1 in vivo. Biochem Biophys Res Commun. 2006;343: 738–744. doi: 10.1016/j.bbrc.2006.03.036 16563347

[8] Gupta A, Madhavan M v., Poterucha TJ, DeFilippis EM, Hennessey JA, Redfors B, et al. Association between antecedent statin use and decreased mortality in hospitalized patients with COVID-19. Nat Commun. 2021;12. doi: 10.1038/s41467-021-21553-1 33637713 PMC7910606

[9] Muchova L, Wong RJ, Hsu M, Morioka I, Vitek L, Zelenka J, et al. Statin treatment increases formation of carbon monoxide and bilirubin in mice: A novel mechanism of in vivo antioxidant protection. Can J Physiol Pharmacol. 2007;85: 800–810. doi: 10.1139/y07-077 17901890

[10] Ayeh SK, Abbey EJ, Khalifa BAA, Nudotor RD, Osei AD, Chidambaram V, et al. Statins use and COVID-19 outcomes in hospitalized patients. PLoS ONE. Public Library of Science; 2021. doi: 10.1371/journal.pone.0256899 34506533 PMC8432819

[11] Fedson DS. Pandemic Influenza: A Potential Role for Statins in Treatment and Prophylaxis. Clinical Infectious Diseases. 2006;43: 199–205. doi: 10.1086/505116 16779747 PMC7107836

[12] Bhattacharya J, Booy R, Casadevall A, dela Cruz C, Fedson DS, Garcia JGN, et al. A practical treatment for COVID-19 and the next pandemic. Pharmacol Res Perspect. 2022. doi: 10.1002/prp2.988 35837790 PMC9284194

[13] Fedson DS, Opal SM, Rordam OM. Hiding in plain sight: An approach to treating patients with severe covid-19 infection. mBio. 2020;11. doi: 10.1128/mBio.00398-20 32198163 PMC7157814

[14] Oskotsky T, Marić I, Tang A, Oskotsky B, Wong RJ, Aghaeepour N, et al. Mortality Risk among Patients with COVID-19 Prescribed Selective Serotonin Reuptake Inhibitor Antidepressants. JAMA Netw Open. 2021. doi: 10.1001/jamanetworkopen.2021.33090 34779847 PMC8593759

[15] Cheng F, Desai RJ, Handy DE, Wang R, Schneeweiss S, Barabási AL, et al. Network-based approach to prediction and population-based validation of in silico drug repurposing. Nat Commun. 2018;9. doi: 10.1038/s41467-018-05116-5 30002366 PMC6043492

[16] Novak R, Lin T, Kaushal S, Sperry M, Vigneault F, Gardner E, et al. Target-agnostic discovery of Rett Syndrome therapeutics by coupling computational network analysis and CRISPR-enabled in vivo disease modeling. bioRxiv. 2022. doi: 10.1101/2022.03.20.485056

[17] Thompson PD, Panza G, Zaleski A, Taylor B. Statin-Associated Side Effects. J Am Coll Cardiol. 2016;67: 2395–2410. doi: 10.1016/j.jacc.2016.02.071 27199064

[18] Naci H, Brugts J, Ades T. Comparative tolerability and harms of individual statins: A study-level network meta-analysis of 246 955 participants from 135 randomized, controlled trials. Circ Cardiovasc Qual Outcomes. 2013;6: 390–399. doi: 10.1161/CIRCOUTCOMES.111.000071 23838105

[19] Kanehisa M, Goto S. KEGG: Kyoto Encyclopedia of Genes and Genomes. Nucleic Acids Res. 2000;28: 27–30. doi: 10.1093/nar/28.1.27 10592173 PMC102409

[20] Han H, Shim H, Shin D, Shim JE, Ko Y, Shin J, et al. TRRUST: A reference database of human transcriptional regulatory interactions. Sci Rep. 2015;5: 1–11. doi: 10.1038/srep11432 26066708 PMC4464350

[21] Han H, Cho JW, Lee S, Yun A, Kim H, Bae D, et al. TRRUST v2: An expanded reference database of human and mouse transcriptional regulatory interactions. Nucleic Acids Res. 2018;46: D380–D386. doi: 10.1093/nar/gkx1013 29087512 PMC5753191

[22] NIH LINCS Program. In: https://lincsproject.org/.

[23] Lamb J, Crawford ED, Peck D, Modell JW, Blat IC, Wrobel MJ, et al. The Connectivity Map: Using Gene-Expression Signatures to Connect Small Molecules, Genes, and Disease. www.broad.mit.edu/cmap. 10.1126/science.1132939 17008526

[24] Suzuki T, Itoh Y, Sakai Y, Saito A, Okuzaki D, Motooka D, et al. Generation of human bronchial organoids for SARS-CoV-2 research Daisuke Motooka 7. bioRxiv. 2020. doi: 10.1101/2020.05.25.115600

[25] Desai N, Neyaz A, Szabolcs A, Shih AR, Chen JH, Thapar V, et al. Temporal and spatial heterogeneity of host response to SARS-CoV-2 pulmonary infection. Nat Commun. 2020;11. doi: 10.1038/s41467-020-20139-7 33298930 PMC7725958

[26] Yang L, Han Y, Nilsson-Payant BE, Gupta V, Wang P, Duan X, et al. A Human Pluripotent Stem Cell-based Platform to Study SARS-CoV-2 Tropism and Model Virus Infection in Human Cells and Organoids. Cell Stem Cell. 2020;27: 125–136.e7. doi: 10.1016/j.stem.2020.06.015 32579880 PMC7303620

[27] Lieberman NAP, Peddu V, Xie H, Shrestha L, Huang ML, Mears MC, et al. In vivo antiviral host transcriptional response to SARS-CoV-2 by viral load, sex, and age. PLoS Biol. 2020;18. doi: 10.1371/JOURNAL.PBIO.3000849 32898168 PMC7478592

[28] Nienhold R, Ciani Y, Koelzer VH, Tzankov A, Haslbauer JD, Menter T, et al. Two distinct immunopathological profiles in autopsy lungs of COVID-19. Nat Commun. 2020;11. doi: 10.1038/s41467-020-18854-2 33033248 PMC7546638

[29] Sharma A, Garcia G, Wang Y, Plummer JT, Morizono K, Arumugaswami V, et al. Human iPSC-Derived Cardiomyocytes Are Susceptible to SARS-CoV-2 Infection. Cell Rep Med. 2020;1. doi: 10.1016/j.xcrm.2020.100052 32835305 PMC7323681

[30] Blanco-Melo D, Nilsson-Payant BE, Liu WC, Uhl S, Hoagland D, Møller R, et al. Imbalanced Host Response to SARS-CoV-2 Drives Development of COVID-19. Cell. 2020;181: 1036–1045.e9. doi: 10.1016/j.cell.2020.04.026 32416070 PMC7227586

[31] Weston S, Coleman CM, Haupt R, Logue J, Matthews K, Li Y, et al. Broad Anti-coronavirus Activity of Food and Drug Administration-Approved Drugs against SARS-CoV-2 In Vitro and SARS-CoV In Vivo. J Virol. 2020;94. doi: 10.1128/JVI.01218-20 32817221 PMC7565640

[32] Sperry MM, Novak R, Keshari V, M Dinis AL, Cartwright MJ, Camacho DM, et al. Enhancers of host immune tolerance to bacterial infection discovered using linked computational and experimental approaches. Advanced Science. 2022. doi: 10.1002/advs.202200222 35706367 PMC9475558

[33] Ayres JS, Schneider DS. Tolerance of infections. Annu Rev Immunol. 2012;30: 271–294. doi: 10.1146/annurev-immunol-020711-075030 22224770

[34] Xing J, Shankar R, Ko M, Zhang K, Zhang S, Drelich A, et al. Deciphering COVID-19 host transcriptomic complexity and variations for therapeutic discovery against new variants. iScience. 2022;25. doi: 10.1016/j.isci.2022.105068 36093376 PMC9439871

[35] Xing J, Paithankar S, Liu K, Uhl K, Li X, Ko M, et al. Published anti-SARS-CoV-2 in vitro hits share common mechanisms of action that synergize with antivirals. Brief Bioinform. 2021;22. doi: 10.1093/bib/bbab249 34245241 PMC8344595

[36] Ward NC, Watts GF, Eckel RH. Statin Toxicity: Mechanistic Insights and Clinical Implications. Circulation Research. Lippincott Williams and Wilkins; 2019. pp. 328–350. 10.1161/CIRCRESAHA.119.315233 31170055

[37] US Food & Drug Administration. FDA Approves First Treatment for COVID-19. In: https://www.fda.gov/news-events/press-announcements/fda-approves-first-treatment-covid-19. 22 Oct 2020.

[38] US Food & Drug Administration. FDA Takes Actions to Expand Use of Treatment for Outpatients with Mild-to-Moderate COVID-19. In: https://www.fda.gov/news-events/press-announcements/fda-takes-actions-expand-use-treatment-outpatients-mild-moderate-covid-19. 21 Jan 2022.

[39] Schultz DC, Johnson RM, Ayyanathan K, Miller J, Whig K, Kamalia B, et al. Pyrimidine inhibitors synergize with nucleoside analogues to block SARS-CoV-2. Nature. 2022;604: 134–140. doi: 10.1038/s41586-022-04482-x 35130559 PMC10377386

[40] Pruijssers AJ, George AS, Schäfer A, Leist SR, Gralinksi LE, Dinnon KH, et al. Remdesivir Inhibits SARS-CoV-2 in Human Lung Cells and Chimeric SARS-CoV Expressing the SARS-CoV-2 RNA Polymerase in Mice. Cell Rep. 2020;32. doi: 10.1016/j.celrep.2020.107940 32668216 PMC7340027

[41] Rosenke K, Hansen F, Schwarz B, Feldmann F, Haddock E, Rosenke R, et al. Orally delivered MK-4482 inhibits SARS-CoV-2 replication in the Syrian hamster model. Nat Commun. 2021;12. doi: 10.1038/s41467-021-22580-8 33863887 PMC8052374

[42] Cox RM, Wolf JD, Plemper RK. Therapeutically administered ribonucleoside analogue MK-4482/EIDD-2801 blocks SARS-CoV-2 transmission in ferrets. Nat Microbiol. 2021;6: 11–18. doi: 10.1038/s41564-020-00835-2 33273742 PMC7755744

[43] Wahl A, Gralinski LE, Johnson CE, Yao W, Kovarova M, Dinnon KH, et al. SARS-CoV-2 infection is effectively treated and prevented by EIDD-2801. Nature. 2021;591: 451–457. doi: 10.1038/s41586-021-03312-w 33561864 PMC7979515

[44] Ahmed MS, Farag AB, Wang P, Boys IN, Menendez-Montes I, Uyen N, et al. Identification of Atovaquone and Mebendazole as Repurposed Drugs with Antiviral Activity against SARS-CoV-2. ChemRxiv. 2021.

[45] Björkhem-Bergman L, Bergman P, Andersson J, Lindh JD. Statin treatment and mortality in bacterial infections—a systematic review and meta-analysis. PLoS ONE. 2010. doi: 10.1371/journal.pone.0010702 20502712 PMC2873291

[46] Jain MK, Ridker PM. Anti-inflammatory effects of statins: Clinical evidence and basic mechanisms. Nature Reviews Drug Discovery. 2005. pp. 977–987. doi: 10.1038/nrd1901 16341063

[47] Broady R, Levings MK. Tuning up transplantation Graft-versus-host disease: suppression by statins. 2008. http://www.nature.com/naturemedicine 10.1038/nm1108-1155 18989280

[48] Fakhouri EW, Peterson SJ, Kothari J, Alex R, Shapiro JI, Abraham NG. Genetic Polymorphisms Complicate COVID-19 Therapy: Pivotal Role of HO-1 in Cytokine Storm. Antioxidants. 2020;9: 636. doi: 10.3390/antiox9070636 32708430 PMC7402116

[49] Bergman P, Linde C, Pütsep K, Pohanka A, Normark S, Henriques-Normark B, et al. Studies on the antibacterial effects of statins—in vitro and in vivo. PLoS One. 2011;6: 1–7. doi: 10.1371/journal.pone.0024394 21912631 PMC3166163

[50] de Spiegeleer A, van Migerode J, Bronselaer A, Wynendaele E, Peelman M, Vandaele F, et al. Statin Intake and All-Cause Mortality among Older Nursing Home Residents. Gerontology. 2022;68: 407–411. doi: 10.1159/000516862 34134106

[51] Rosenson RS. Statins: Actions, side effects, and administration. UpToDate. Waltham, MA: Wolters Kluwer Health; 2021.

