# Supplementary material for: Target-agnostic drug prediction integrated with medical record analysis uncovers differential associations of statins with increased survival in COVID-19 patients
Source: PLoS Comput Biol. 2023 May 5;19(5):e1011050. doi: 10.1371/journal.pcbi.1011050 (PMC10191356; doi:10.1371/journal.pcbi.1011050)
Supplement: S1 Form — If you are a user from an academic or non-profit research institution, please fill out this form to obtain access to NeMoCAD. (DOCX) [file pcbi.1011050.s002.docx]

If you are a user from an academic or non-profit research institution, please fill out the form below to download or to obtain access to “NeMoCAD,” the inference and modeling code described in Sperry et al. *PLoS Comput. Biol.* 2023.

**If you are seeking a Commercial Use license to NeMoCAD, please contact the Office of** **Technology Development at Harvard University to discuss licensing options.**

Last Name:

First Name:

Title:

Department:

Institution:

Email:

Country:

Signature Attesting to Agreement to End User License Agreement

Name Date

**All fields are required.**

INSTRUCTIONS FOR DOWNLOADING NeMoCAD:

- Submit the S1 Form to Paula Murphy (paula.murphy@wyss.harvard.edu).
- Request access via Zenodo: Sahil Loomba, Shruti Kaushal, Megan Sperry, & Richard Novak. (2023). NeMoCAD: Network Models for Causally Aware Discovery (1.0.1-2). Zenodo. <https://doi.org/10.5281/zenodo.7692712>
- After approval, access NeMoCAD for download on Zenodo.

Please direct any questions to:

**Paula Murphy**| **Senior Director, Research Administration & Faculty Affairs**
Wyss Institute for Biologically Inspired Engineering at Harvard University
3 Blackfan Circle, 1^st^ Floor, Boston, MA 02115

paula.murphy@wyss.harvard.edu

**Non-Commercial Research and Academic Use Software License and Terms of Use**

NeMoCAD is a software package that includes original code created by the Harvard researchers listed below (the “Software”), and third-party code that may be obtained by End Users separately. The Software is designed to for discovering biological mechanisms and drug therapies given transcriptomics data. The Software was developed by Sahil Loomba, Shruti Kaushal, Megan Sperry, and Richard Novak at Harvard University. It is distributed for free academic and non-commercial research use by the President and Fellows of Harvard College (“Harvard”).

Using the Software indicates your agreement to be bound by the terms of this Software Use Agreement (“Agreement”). Absent your agreement to the terms below, you (the “End User”) have no rights to hold or use the Software whatsoever.

Harvard agrees to grant hereunder a limited non-exclusive license to End User for the use of the Software in the performance of End User’s internal, non-commercial research and academic use at End User’s academic or not-for-profit research institution (“Institution”) on the following terms and conditions:

1. **NO REDISTRIBUTION.** The Software remains the property of Harvard, and End User shall not publish, distribute, or otherwise transfer or make available the Software to any other party.

2. **NO COMMERCIAL USE.** End User shall not use the Software for Commercial use and any such use of the Software is expressly prohibited. “Commercial use” includes, but is not limited to, (i) use of the Software in fee-for-service arrangements, (ii) use of the Software by core facilities or laboratories to provide research services to (or in collaboration with) for-profit third parties for a fee, and (iii) use of the Software in industry-sponsored and/or collaborative research projects in which any commercial rights are granted to the sponsor or collaborator. If End User wishes to use the Software for Commercial use, End User must execute a separate license agreement with Harvard.

Requests for use of the Software for Commercial use, please contact:

Office of Technology Development

Harvard University

Smith Campus Center, Suite 727E

1350 Massachusetts Avenue Cambridge, MA 02138 USA Telephone: (617) 495-3067

[E-mail: otd@harvard.edu](mailto:otd@harvard.edu)

3. **OWNERSHIP AND COPYRIGHT NOTICE.** Harvard owns all intellectual property in the Software. End User shall gain no ownership to the Software. End User shall not remove or delete, and shall retain in the Software (including in any modifications to the Software and in any Derivative Works), the copyright, trademark, or other notices pertaining to Software as provided with the Software.

4. **DERIVATIVE WORKS.** End User may create and use Derivative Works, as such term is defined under U.S. copyright laws, provided that any such Derivative Works shall be restricted to non-commercial, internal research and academic use at End User’s Institution. End User may not distribute Derivative Works to any third parties.

5. **FEEDBACK.** In order to improve the Software, comments from End Users may be useful. End User agrees to provide Harvard with feedback on the End User’s use of the Software (e.g., any bugs in the Software, the user experience, etc.). Harvard is permitted to use such information provided by End User in making changes and improvements to the Software without compensation or accounting to End User.

6. **NON ASSERT.** End User acknowledges that Harvard may develop modifications to the Software that may be based on the feedback provided by End User under Section 5 above. Harvard shall not be restricted in any way by End User regarding its use of such information. End User acknowledges the right of Harvard to prepare, publish, display, reproduce, transmit and or use modifications to the Software that may be substantially similar or functionally equivalent to End User’s modifications and/or improvements if any. In the event that End User obtains patent protection for any modification or improvement to Software, End User agrees not to allege or enjoin infringement of End User’s patent against Harvard, or any of the researchers, medical or research staff, officers, directors and employees of those institutions.

7. **PUBLICATION & ATTRIBUTION.** End User has the right to publish, present, or share results from the use of the Software. In accordance with customary academic practice, End User will acknowledge Harvard as the provider of the Software and may cite the relevant reference(s) from the following list of publications:

Sperry, M. M., Oskotsky, T., Marić, I., Kaushal, S., Takeda, T., Horvath, V., ... & Novak, R. (2023). Target-agnostic drug prediction integrated with medical record analysis uncovers differential associations of statins with increased survival in COVID-19 patients. *PLoS Comput. Biol*.

Novak, R., Lin, T., Kaushal, S., Sperry, M., Vigneault, F., Gardner, E., ... & Ingber, D. E. (2022). Target-agnostic discovery of Rett Syndrome therapeutics by coupling computational network analysis and CRISPR-enabled in vivo disease modeling. *bioRxiv*, 2022-03.

8. **NO WARRANTIES.** THE SOFTWARE IS PROVIDED "AS IS." TO THE FULLEST EXTENT PERMITTED BY LAW, HARVARD HEREBY DISCLAIMS ALL WARRANTIES OF ANY KIND (EXPRESS, IMPLIED OR OTHERWISE) REGARDING THE SOFTWARE, INCLUDING BUT NOT LIMITED TO ANY IMPLIED WARRANTIES OF MERCHANTABILITY, FITNESS FOR A PARTICULAR PURPOSE, OWNERSHIP, AND NON-INFRINGEMENT. HARVARD MAKES NO WARRANTY ABOUT THE ACCURACY, RELIABILITY, COMPLETENESS, TIMELINESS, SUFFICIENCY OR QUALITY OF THE SOFTWARE. HARVARD DOES NOT WARRANT THAT THE SOFTWARE WILL OPERATE WITHOUT ERROR OR INTERRUPTION.

9. **LIMITATIONS OF LIABILITY AND REMEDIES**. USE OF THE SOFTWARE IS AT END USER’S OWN RISK. IF END USER IS DISSATISFIED WITH THE SOFTWARE, ITS EXCLUSIVE REMEDY IS TO STOP USING IT. IN NO EVENT SHALL HARVARD BE LIABLE TO END USER OR ITS INSTITUTION, IN CONTRACT, TORT OR OTHERWISE, FOR ANY DIRECT, INDIRECT, SPECIAL, INCIDENTAL, CONSEQUENTIAL, PUNITIVE OR OTHER DAMAGES OF ANY KIND WHATSOEVER ARISING OUT OF OR IN CONNECTION WITH THE SOFTWARE, EVEN IF HARVARD IS NEGLIGENT OR OTHERWISE AT FAULT, AND REGARDLESS OF WHETHER HARVARD IS ADVISED OF THE POSSIBILITY OF SUCH DAMAGES.

10. **INDEMNIFICATION.** To the extent permitted by law, End User shall indemnify, defend and hold harmless Harvard, their corporate affiliates, current or future directors, trustees, officers, faculty, medical and professional staff, employees, students and agents and their respective successors, heirs and assigns (the "Indemnitees"), against any liability, damage, loss or expense (including reasonable attorney's fees and expenses of litigation) incurred by or imposed upon the Indemnitees or any one of them in connection with any claims, suits, actions, demands or judgments arising from End User’s breach of this Agreement or its Institution’s use of the Software except to the extent caused by the gross negligence or willful misconduct of Harvard. This indemnification provision shall survive expiration or termination of this Agreement.

11. **GOVERNING LAW.** This Agreement shall be construed and governed by the laws of the Commonwealth of Massachusetts regardless of otherwise applicable choice of law standards.

12. **NON-USE OF NAME.** Nothing in this License and Terms of Use shall be construed as granting End Users or their Institutions any rights or licenses to use any trademarks, service marks or logos associated with the Software. End User may not use the terms “Harvard” (or a substantially similar term) in any way that is inconsistent with the permitted uses described herein. End Users may not use any name or emblem of Harvard or any of its schools or subdivisions for any purpose, or to falsely suggest any relationship between End User (or its Institution) and Harvard, or in any manner that would infringe or violate any of its rights.

13. End User represents and warrants that it has the legal authority to enter into this License and Terms of Use on behalf of itself and its Institution.

***
